# Targeting DNA Topoisomerase II in Antifungal Chemotherapy

**DOI:** 10.3390/molecules27227768

**Published:** 2022-11-11

**Authors:** Kavya Kondaka, Iwona Gabriel

**Affiliations:** Department of Pharmaceutical Technology and Biochemistry, Gdansk University of Technology, 80-233 Gdansk, Poland

**Keywords:** antifungals, yeast topoisomerase 2, inhibitor, differential sensitivity, potential drugs

## Abstract

Topoisomerase inhibitors have been in use clinically for the treatment of several diseases for decades. Although those enzymes are significant molecular targets in antibacterial and anticancer chemotherapy very little is known about the possibilities to target fungal topoisomerase II (topo II). Raising concern for the fungal infections, lack of effective drugs and a phenomenon of multidrug resistance underlie a strong need to expand the range of therapeutic options. In this review paper, we discussed the usefulness of fungal topo II as a molecular target for new drug discovery. On the basis of previously published data, we described structural and biochemical differences between fungal and human enzymes as well as a molecular basis of differential sensitivity to known anticancer drugs targeting the latter. This review focuses especially on highlighting the differences that may underlie the selectivity of action of new inhibitors. Distinct sites within fungal topo II in comparison with human counterparts are observed and should be further studied to understand the significance of those sites and their possible usage in design of new drugs.

## 1. Introduction

Fungal infections are of serious medical concern, especially in patients suffering with immunosuppressive diseases [1]. There is a need of appropriate diagnosis as those that go undiagnosed at the initial stage may lead to severe acute diseases and thus open a pathway for other microbial infections and bacterial attacks [2]. It is of immense need to identify targets that are both fungal-specific and suitable for developing drugs. Anti-fungal agents such as Amphotericin B are highly effective but have various cons with over usage such as causing nephrotoxicity, poor oral activity, poor pharmacokinetics, emergence of multi-drug resistance strains and genotoxicity [3]. Amphotericin B and voriconazole exhibit differential fungicidal and/or fungistatic roles against various strains of *Aspergillus* fungi thus indicating the differential effect of these compounds on its growth and inhibition at lower and higher concentrations. Some antifungal agents like azoles and flucytosine showed fungistatic activity by blocking the growth at a lower concentration than that for fungicidal activity against *C. albicans* [4,5] Alternative antifungal targets are needed so as to expand the range of therapeutic options, especially the enzymes that are important for the biosynthesis of proteins, amino acids or DNA processing [6,7,8]. Additionally, invasive fungal infections complicate the clinical course of COVID-19 and are associated with a significant increase in mortality, especially in critically ill patients admitted to an intensive care unit [9]. Thus, with the current massive increases in drug-resistant microbial infection as well as a significant role of fungal infections in the death toll of COVID-19, discovering new antifungal compounds is extremely important.

DNA topoisomerases are a set of ubiquitous enzymes that play a crucial role in the control and proper regulation of topology during cellular events such as DNA replication, repair, chromosome segregation and condensation, thus responsible for cell viability [10,11,12,13,14,15,16,17]. Inhibition of topoisomerase II by a drug-stabilized-DNA complex leads to the arrest of replication fork progression and formation of double strand breaks (DSBs) [18]. Topoisomerases are classified into type I and II depending upon the catalyzation and re-ligation of either a single strand or double strands DNA breaks, respectively. Type I is subcategorized into IA, IB and IC whereas, type II is further classified into IIA (DNA gyrase and top IV in prokaryotes; eukaryotic type II) and IIB (top VI) [19,20]. Topoisomerases are the targets for various antimicrobial and anticancer agents and are considered to be crucial to inhibit them for the suppression of their activity [21,22,23,24,25,26,27,28,29,30,31].

**Figure 1:**
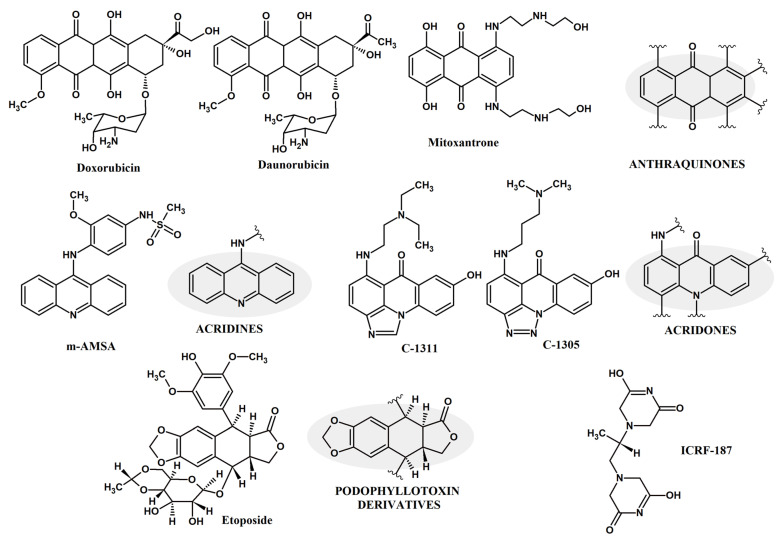
Selected compounds that either act as intercalators or interrupting agents to human topoisomerase II from the group of anthraquinone, acridine, acridone or podophyllotoxin derivatives and ICRF-187.

There have been several drugs existing for decades that either act as intercalators or interrupting agents to human topo II (HsTopo II) enzymes and this led researchers to decipher the mechanism involved in choosing them as the drug targets (Figure 1). 

For example, doxorubicin, etoposide, teniposide, idarubicin, epirubicin and mitoxantrone are FDA approved medicines [32]. The mechanism of action of the drugs that target topoisomerase enzymes is by stabilizing the cleaved enzyme-DNA complex and thus, converting the enzyme into a cellular poison. Anticancer drugs’ mode of action is mainly related to increasing covalent-topo II-cleaved DNA complexes level. Increased levels of topo II can alter the genomic integrity and leads to drug hypersensitivity; while decreased levels lead to drug resistance [20,33]. Eukaryotic topoisomerase II (topo II) are structurally similar and organized similarly to that of bacterial enzymes DNA gyrase (top IIA) and topo IV [34,35,36]. As fungal topoisomerase II is considered, its function is required for the segregation of daughter molecules at the termination of DNA replication and is essential for proper growth [37]. Thus, novel anti-fungal drugs can be proposed that can also inhibit the fungal DNA topoisomerases activity.

There are two categories of compounds based on the mechanism of topo II inhibition called catalytic inhibitors and poisons. The first one decreases the overall activity of the enzyme and the latter is involved in increasing the levels of topo II-DNA-cleaved complexes [38]. Topo II poisons differ from each other. Etoposide, teniposide and the DNA intercalators doxorubicin, daunorubicin, amsacrine (m-AMSA) inhibit DNA re-ligation, whereas the others, such as quinolone CP-115,193, the ellipticines, azatoxins and genistein, enhance the formation of topo II cleavage complexes [27]. For drugs such as m-AMSA, C-1305, C-1311 DNA intercalation properties are important steps for poisoning [39,40,41,42,43,44]. Catalytic inhibitor ICRF-187 blocks ATP hydrolysis and inhibits the reopening of the ATPase domain of topo II, thus trapping topological complexes with DNA inside the enzyme [27].

## 2. Role and Structure of Fungal Topoisomerase II

Topo II from *S. cerevisiae* (ScTopo II) was reported as essential for viability [45], whereas topo I was not [46]. ScTopo II is a homo dimer and plays significant roles like relaxing positive and negative supercoils, decatenation and unknotting DNA, in an ATP- and Mg^+2^-dependent manner [17,19,45,47,48]. Apart from these general tasks, topo II participates in more specialized functions which includes supporting the transcription of long genes (>3 kb) which cannot be rescued by topo I. It plays a significant role in the relief of torsional strain during DNA replication by relaxing the positive supercoils ahead of the fork. Its absence exhibited the prevention of mitosis [19]. In budding yeast, topo II regulates the expression of specific gene subsets. A subset of genes are directly up- and down- regulated by topo II inactivation in *S. cerevisiae* [49]. The upregulated subset is poor in essential genes but enriched in TATA-containing genes, e.g., responsible for membrane transport of polyamines. Downregulated genes are essential and related to the remodeling of chromatin and transcriptional regulation [50].

**Figure 2:**
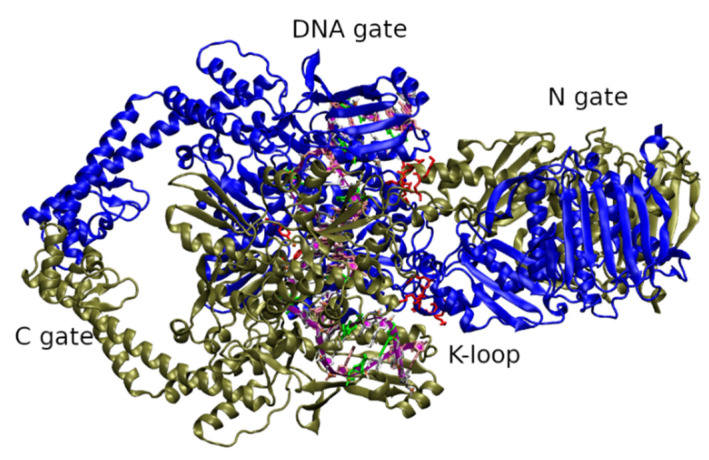
ScTopo II (PDBID: 4GFH) domain organization, three major gates: N-gate, DNA gate and C-gate. Each of the dimer is represented in either blue color or gray color for distinction (a). A strand of DNA, G-segment, is bound at the DNA binding gate (represented in purple ribbon), (b) A consensus K-loop in red licorice representation.

As far as the 3D structure of fungal topoisomerase II is concerned, the only known are those from *S. cerevisiae*: ScTopo II in complex with DNA and a nonhydrolyzable ATP analog (PDBID: 4GFH) (Figure 2) [51], ATPase region (PDBID: 1PVG) and ATP region in complex with ICRF-187 inhibitor (PDBID: 1QZR) [34], a complex between the DNA-binding and cleavage core of ScTopo II and a gate-DNA segment (PDBID: 2RGR) [52]. The structure of the DNA-binding and cleavage core of ScTopo II covalently linked to DNA through its active-site tyrosine is also published (PDBID: 3L4J and 3L4K metal bound) [53]. Important ScTopo II residues starting from the TOPRIM domain till the C-gate were also identified and recorded as PDBID: 1BJT and 1BGW which are responsible for the ATP-dependent strand breakage, passage rejoining dsDNA [54,55].

The domain organization in ScTopo II (Figure 2) basically has three main domains: (a) ATPase domain (residues 1–408) consisting of N-gate interface and a transducer region namely, K-loop; (b) DNA binding/cleavage domain (DNA-gate) (residues 410–1200) having TOPRIM domain and winged helical domain and (c) C-terminal region/C-gate (residues up to 1418). Studies revealed that this enzyme bound to a 30 bp G-segment showed A-form DNA that is bent to 150°. This deformation has a significant role in cleavage and correct positioning of the DNA backbone within the active site [19].

The topological re-arrangements by the ScTopo II enzyme are a multi-stage mechanism known as strand passage mechanism and involves a controlled association and dissociation of the aforementioned 3 distinct subunit dimerization interfaces, hence guiding the physical movement of one DNA duplex through another (Figure S1). 

The first step includes binding of DNA through N-gate and G-segment bending (angle 150°) at the DNA gate (Figure S1 step 1). The next step includes dimerization of the ATPase subunits in the presence of ATP to capture the second DNA duplex namely, the transport segment (T-segment). (Figure S1 step 2). A cascade of conformational changes is triggered; thus, forming a transient double stranded break (DSB) by cleaving the G-segment. The DSB is catalyzed by a pair of symmetrically related tyrosines (active site at residue 782), in conjunction with a Mg^+2^ ion-binding TOPRIM fold forming a transient covalent “topo II-DNA-cleaved complex” via a nucleophilic attack (Figure S1 step 3,4) [56]. The T-segment transport through the break in the G-segment occurs simultaneously with the hydrolysis of one ATP molecule (Figure S1 step 5). The catalytic cycle ends with re-ligation of the G segment and hydrolysis of another ATP molecule, which induces reopening of the C gate, transport of the T segment through the C gate, and finally opening of the enzyme’s N gate (Figure S1 step 6,7). Further, the enzyme is reset for the next cycle (Figure S1, step 8). [51,54,57,58].

In-depth analysis of crystal structures determined for yeast and HsTopo II α and β indicates that both enzymes are almost identical with respect to ATP binding as well as active sites (Table 1). This is a normal situation because evolutionarily only those regions that are not crucial for catalysis could be variable. Nevertheless, the differences in the protein sequences of topo II enzyme in fungal and human counterparts was observed using Constraint-based Multiple Alignment Tool in NCBI and the key sites along with their function are identified and listed in Table 1 and Figure S2.

As can be seen in Table 1 and Figure S2, TOPRIM domain as well as a region responsible for DNA interaction are not significantly conserved. Importantly, it was reported that fungal and mammalian as well as other eukaryotic topoisomerases exhibit structural differences, e.g., within so called K-loop, which is important for DNA strand passage activities. Schmidt et al., had also reported the differences in K-loop region of various eukaryotic type II A topoisomerases where fungal enzymes bears six lysines in K-loop and all other eukaryotic type II possess three, two of which are conserved from yeast to humans [51].

Two of the other most significant differences between HsTopo IIα and ScTopo II were also identified in the literature. The first is located in the TOPRIM region and the second in the C-gate. Fungal enzyme differs from human one by the lack of important interactions in the TOPRIM domain between a flexible “Greek-key” element and residues present in TOPRIM insert of HsTopo IIα (helix α11, residues 670–686). These interactions are important in respect of contact with partner subunit of dimeric human enzyme. The second difference maps to the C-gate, where a short α-helix in human topo IIα (residues 1086–1092) replaces a β-hairpin in the yeast model, thereby forming an interface with one of the two long α-helical arms (residues 1015–1060) that extend from the DNA gate. The functional significance of these modifications, if any, is not known at present [61].

Structural differences between fungal and mammalian topoisomerases are also identified by homology-based modeling of ScTopo II performed with the program MODELLER based on the sequence alignment of PDB ID 4GFH [64]. The finalized structural model of topo II was accomplished by employing Loop Refine in MODELLER and utilizing as the modeling template along with the 17 residues refined in between residue numbers 251 to 281, a further 12 more amino acids residues in between 401 and 421, which are considered to be the most important and longest missing residue sequence in the complex. These two missing residue sequences are closer to DNA. Furthermore, these missing sequences are quite different from HsTopo IIα and HsTopoIIβ.

Additionally, we performed an alignment to observe the major differences in the protein sequences of topo II enzymes in three different fungal strains *Candida albicans* (Uniprot ID P87078), *Candida glabrata* (Uniprot ID O93794) and *Saccharomyces cerevisiae* (Uniprot ID P06786) with that of both human isoforms, i.e., Topo IIα (Uniprot ID P11388) and Topo IIβ (Uniprot ID Q02880) (Figure S3). For this, Uniprot ID’s were collected and FASTA sequences were multi-aligned using Clustal Omega tool in Jalview software which was further analyzed for visualization and analysis [64]. The sequence alignment showed both the similarities as well as differences in certain residues. Taking ScTopo II enzyme as the reference for numbering the amino acids, the similarities are marked with bold red rectangular bars in Figure S3, indicating ATP nucleotide binding region (residues 140–147 & 365–367), the K-loop region (residues 333–338), metal binding sites (residues 449, 526, 528), active site (residue 782), intercalator and bending to DNA (residue 833), and the region that interacts with DNA (residues 965–974). Similarly, presence of residues in the position 257–267 only in fungal strains and absence of amino acid residues in between the regions 660–664 (corresponding to helix α11), sites 919 and 940 in all three fungal strains could be of great importance in terms of therapeutic use (represented in blue rectangular bars in Figure S3). These might be predicted as the possible potential targets for the study. The residue numbers are with reference to the ScTopo II enzyme.

### 2.1. Fungal Topoisomerase II Inhibition

A study conducted by Dinardo et al., showed that a mutation in *S. cerevisiae TOPO2* gene is responsible for both temperature-sensitive growth and altered topo II enzymatic activity. Temperature sensitive (ts) mutant growth and enzymatic activity during the termination of DNA replication, especially the segregation of daughter chromosomes was inhibited. At a non-permissive temperature, cells undergo only one round of DNA replication and get arrested at medial nuclear division. Accumulation of DNA in the form of highly intertwined catenated dimers were also observed after one cycle of DNA replication, since there was no disjunction of the sister chromatids in the absence of topo II activity [37]. The results therefore indicate the conclusion that topoisomerase II activity is essential for *S. cerevisiae* growth and targeting fungal enzyme is worth considering.

Following is some of the noteworthy cases/mechanisms reported where fungal topo II can be inhibited in the presence of drugs, which are further explained in detail:

- At the ATP domain.
- After 1st ATP hydrolysis and before 2nd ATP hydrolysis.
- Inhibition of DNA DSBs re-ligation at the catalytic domain.
- De novo duplications at the DSBs using Non-Homologous End Joining (NHEJ) repair pathway.
- Blocking active catalytic tyrosine.
- Preventing sister chromatids segregation at termination of DNA replication.

Topoisomerase II targeting by benzoquinoline derivatives and their antifungal properties better than control drug nystatin [65] are promising results and support the idea of this enzyme’s usefulness for new drug discovery. Furthermore, phenolic compounds from pomegranate exhibited strong topoisomerase inhibitory potential as well as antifungal activity. Punicalagin showed strong inhibitory activities against both topo I and topo II of *C. albicans* with the IC_50_ values of 9 µM and 4.6 µM, respectively. Similarly, punicalin inhibited type I and II with IC_50_ values of 14.7 µM and 40.7 µM whereas ellagic acid showed lowest activity with estimated IC_50_ values 56.6 µM and 53.9 µM, respectively. Punicalagin displayed the most promising antifungal activity towards the pathogens considered in this work [66].

Known anticancer compounds like m-AMSA (acridine-compound), etoposide and its derivatives such as A-80198 and A-75272 (a tricyclic quinolones) also proved the differences in biochemical properties and in sensitivity to inhibitors of fungal and human enzymes. m-AMSA has a moderate inhibitory effect and less sensitivity, i.e., four-fold lower in magnitude against fungal topo II than in the human counterpart was demonstrated [11]. Compounds A-80198 and A-75272 were also analyzed in relation to inhibitory effects against fungal and calf thymus topoisomerase II. The first one similar to etoposide exhibited greater efficiency against mammalian enzyme; however, the *C. albicans* topo II was found to be most sensitive to the second derivative of quinolone, A-75272 [67]. Interestingly, the differences in sensitivity to the action of inhibitors do not depend on the mechanism of action. m-AMSA as well as etoposide act as topoisomerase II “poisons”, whereas ICRF-187 is a catalytic inhibitor and acts as an inhibitor of ATPase activity not only of fungal enzyme, but of both HsTopo IIs as well. However, it was demonstrated that ICRF-187 is much more active against both human topo II isoforms (Topo IIα and Topo IIβ) than against budding yeast topo II (ScTopo II), where the mechanism of action is to stabilize the ATP-dependent dimerization interface. Similarly, differential sensitivity to resveratrol, a polyphenolic compound found in various plant sources has also been shown, despite the fact that it has a different mechanism of action and inhibits topo II by preventing the ATPase domain dimerization rather than stabilizing it [68]. The inhibitory effect of another known topoisomerase II, inhibitors C-1311 and C-1305, on human and yeast enzymes was also analyzed. C-1311 as well as C-1305 totally inhibited ScTopo II-mediated relaxation at a concentration 50 μM. As previously reported, C-1305 diminished the relaxation reaction by 50% at a concentration of 2.5 μM and totally inhibited human topoisomerase II-mediated relaxation at 10 μM. The same level of HsTopo II inhibition ability was determined for C-1311 (IC_50_ 2.5 μg mL^−1^; 6.5 μM). So human enzymes are much more sensitive [69].

### 2.2. Molecular Basis of Differential Sensitivity of Fungal Topoisomerase II to Anticancer Drugs

The differential responses of topo II enzymes to some compounds and the great diversity in terms of molecular and structural properties in humans and fungal organisms have demonstrated that these can be used as potential targets for the development of novel anti-fungal agents [11,70,71]. Thus, discovering what the molecular basis of differential sensitivity is seems to be very valuable.

**Figure 3:**
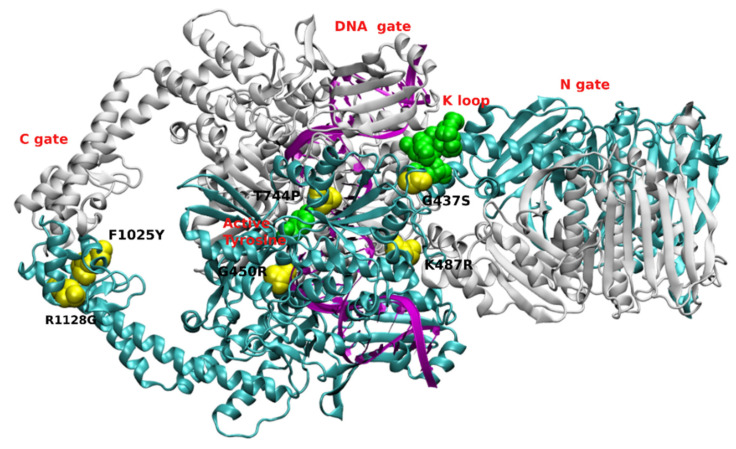
Pictorial representation of selected single mutations (yellow spheres) in yeast topo II enzyme (PDBID: 4GFH) spotted in both ATP binding domain and DNA cleavage/re-ligation domain. Green spheres represent catalytic tyrosine and K-loop. Each of the dimers is represented in either gray color or cyan color for distinction with DNA as violet ribbon.

Previously published results obtained with selected *S. cerevisiae* mutants with improved cell wall permeability showed an inhibitory effect on both type I and type II topoisomerases based on the drug effectiveness with drugs like m-AMSA, etoposide and camptothecin [11]. Mutant cells or mutant enzymes were also studied to analyze which residue in amino acid sequence is responsible for sensitivity or resistance to known topo II inhibitors using ScTopo II as a model enzyme [33,72,73,74]. Mutations are found in the most important parts of the enzyme: ATP binding domain, DNA cleavage/re-ligation site or C-gate. Selected mutation sites are presented in Figure 3.

The results of the mutation studies performed by Elsea [73] indicated that several regions of the yeast topoisomerase II (ScTopo II) protein are important for the action of drugs that stabilize cleavage by the enzyme. Most of the mutations analyzed resulted in resistance to topoisomerase II-targeting drugs although a mutation of particular interest Ser741 to Trp resulted in hypersensitivity to etoposide but did not alter sensitivity to other agents such as m-AMSA. Thus, it suggested that this mutation defines a site on the yeast Topo II that is involved in drug–protein interactions.

Overexpression of yeast top2 alleles [top2-F1025Y, R1128G (top2-FY, RG)] elevates homologous recombination and spontaneous mutation which stabilized the cleavage intermediate in vitro. Studies conducted by Stantial et al. [74] showed the unique mutation signature, i.e., de novo duplications is dependent on NHEJ pathway of DSBs repair. Similar observations were found when a wild-type yeast strain was treated with etoposide, thus stabilizing the topoisomerase II cleavage complex. An in vivo complex of enzyme assay showed a strong signal with the double mutant allele, and detected an enzyme-DNA covalent complex even in the absence of etoposide. Mutated ScTopo II (F1025Y, R1128G) had thus resulted as a self-poisoning enzyme and can be inferred that the top2 dependent mutagenesis has a crucial role to play in genome evolution as well as in obtaining genetic stability in chemotherapy. Further, both these mutant enzymes showed similarity to wild-type ScTopo II in terms of overall catalytic activity in relaxation assays, which lead researchers to conduct cleavage assay in the presence and absence of the topo II-targeting agents. In the absence of inhibitors, both the mutants showed linear fragments with a little amount of purified protein, whereas similar results were absent in the case of wild-type ScTopo II even at higher concentrations of the same. Similar assay conducted in the presence of etoposide and m-AMSA resulted in higher levels of linear DNA with proportional increase in drug concentration, indicating that the purified proteins are intrinsically hypersensitive to m-AMSA and etoposide in vivo. Cleavage assays detected linearized DNA with mutant proteins (R1128G and F1025Y, R1128G) but not with wild-type ScTopo II thus indicating that ATP is not required by the mutant proteins and progression through topoisomerase II catalytic cycle is not required for the drug-independent cleavage of DNA by the proteins.

Topoisomerase II encoding gene mutations in yeast cells (top2-P473L or G737V) showed hypersensitivity to inhibition by m-AMSA and increased the levels of m-AMSA-induced enzyme-DNA covalent complexes [75]. These mutants showed only minor resistance to etoposide. The G737V allele was highly resistant to CP-115,953, whereas the P473L allele sensitivity to this agent was similar to wild-type. Both mutations are in close proximity to that of the catalytic tyrosine and simultaneous presence of these mutations can prevent re-ligation by displacing the active tyrosine further from the DNA and thus reduce its ability to cleave DNA. P437L mutation played a crucial role in deciphering the mechanism involved in ATP-dependent DNA cleavage. For 737 amino acid change to the hydrophobic residue was proposed to be the cause for sensitivity to m-AMSA and not the loss of glycine. Wild-type topoisomerase II showed no inhibition of decatenation assay even at a higher concentration of m-AMSA (100 µg/mL), whereas this assay was inhibited in both mutants proteins with an IC_50_ of 10 µg/mL m-AMSA. ScTopo II-P473L enzyme exhibited increased levels of ATP-independent DNA cleavage complexes in the presence of m-AMSA, thus showing the drug sensitivity [76].

A study conducted by Lee et al. [68] has depicted preferential selectivity for human over yeast topo II enzymes for resveratrol and ICRF-187. Both compounds were more active against the human topo II isoforms (TopIIα and β) than against budding yeast topo II. It is important to note that ICRF-187 stabilizes topo II ATPase domain dimerization, whereas resveratrol impedes this interaction. The assessment of supercoiling relaxation studies was observed in ICRF-187 and resveratrol while comparing with the etoposide which is a topo II poison and observed that these are not poisons rather they inhibit the supercoiling relaxation in a different mode of action than the positive control etoposide. ICRF-187 showed an uncompetitive mode of inhibitory action by decreasing both the V_max_ and K_m_, whereas a mixed-type inhibition was shown by the resveratrol with an increase in K_m_ and less than 2-fold decrease in V_max_. While comparing resveratrol inhibition in human isoforms with ScTopo II enzyme, HsTopo IIα has stronger V_max_ and HsTopo IIβ has a stronger K_m_ [68]. This differential sensitivity suggests that ScTopo II may not be an ideal model to study drugs aimed at targeting the ATPase domain of human topo II. On the other hand, in terms of targeting fungal enzyme results obtained are valuable.

Mutation studies conducted by Sabourin et al. [33] at residue 437 (ATP binding site) of yeast topoisomerase II from a nonpolar Gly to polar Ser (top2G437S) conferred cellular resistance to anticancer drugs; though the purified mutant enzyme ScTopo II-G437S was hypersensitive to anti-cancer agents like CP-115,953, m-AMSA, etoposide in the absence of ATP. However, in the presence of ATP, the enzyme lost its hypersensitivity to anticancer agents and displayed wild-type sensitivity, suggesting a conformational change in the region of residue 437 upon binding of the high energy cofactor. Further analysis indicated that in vivo resistance was caused by decreased levels of active enzyme, while in vitro hypersensitivity reflected (at least in part) an increased enzyme affinity for drugs.

Single point mutation of ScTopo II at site 1012 from a basic residue H to polar Y (DNA gate/gyrA homology domain) showed decrease in its activity in comparison to the wild-type enzyme. This might be due to a decrease in affinity for the DNA whereas the affinity for ATP remained similar. The mutant enzyme showed resistance to CP-115,953 and etoposide; the same as wild-type enzyme sensitivity to m-AMSA and hypersensitivity to ellipticine, a DNA intercalator. It is plausible to say that the mutant enzyme could be able to distinguish the intercalative and non-intercalative agents [73].

Another mutant study in the CAP homology domain (critical role in DNA binding) of yeast topoisomerase II showed hypersensitivity of mutant strain overproducing ScTopo II-T744P enzyme to an intercalator m-AMSA and a non-intercalator CP-115,953; but not to etoposide as there are no differences in minimum lethal concentrations with etoposide in comparison to the wild-type (WT) strain. Their study suggests that the hypersensitivity is definitely not due to the enhanced stability of the T744P protein. Relaxation and decatenation of kinetoplast DNA (kDNA) assays showed similar results for both the mutant and the WT enzyme. Drug-independent DNA cleavage for ScTopo II-T744P enzyme was found to be similar to WT. There were no significant differences in the drug-independent cleavage, whereas the presence of drugs showed enhanced cleavage activities. The level of enhancement is drug concentration dependent. Enhanced cleavage can be clearly seen at low m-AMSA concentrations, whereas there is less difference between the two proteins at higher drug concentrations. A similar pattern was observed with CP-115,953. Etoposide showed no differences in this cleavage assays which confirms the in vivo results. Results performed for mitoxantrone and fluoroquinolones ciprofloxacin and norfloxacin and the quinolone oxolinic acid also indicate that T744P mutant enzyme is also hypersensitive to multiple classes of intercalating agents and fluoroquinolones but not to all classes of topoisomerase II poisons. Researchers also pointed out that the mutant enzyme hypersensitivity was not associated with more stable covalent complexes as it reversed with heat in the presence of m-AMSA, CP-115,953 and etoposide. This results are in contrast with the results obtained for S740W mutant ScTopo II which showed an association between etoposide hypersensitivity and more stable covalent complexes [77]. Molecular modeling indicated that WT enzyme had a distance of 3.97Å between the amino acids 781 and 782, whereas mutant T744P showed an increase in this distance to 9.97Å. Thus, authors concluded that mutation plays important roles in catalysis by yeast topoisomerase II. Other reported mutations such as Gln 743 to Pro showed mutant cells resistance to CP-115,953 and m-AMSA [76].

### 2.3. The Effect of Known Human Topoisomerase Inhibitors on the Growth of Fungal Cells

The human and yeast topoisomerase differ in their sensitivity to known topo II inhibitors and this can be of great importance to choose the fungal enzyme as targets for drugs. The effect of inhibitors from the group of acridine, podophyllotoxin as well as anthracycline derivatives on the growth of fungal cells was also reported [71,72,73,78]. Choosing acridines as one of the molecular targets for development of drugs against bacteria, malarial pathogens and anti-cancer therapy is due to their ability to intercalate into DNA and affect topoisomerases by stabilizing the enzyme–DNA cleavage complex [77,79,80,81]. Most of the derivatives of acridine showed antifungal activity with DNA intercalation as the probable mode of action and are not fungal specific [82,83,84]. Acriflavine is a fungicide, it induces mutations in *S. cerevisiae* and causes cell death in *C. utilis*. Quinacrine showed several independent mechanisms to act against *Candida* cells. Firstly, it showed inhibition in yeast-to-mycelia formation; secondly, it showed antibiofilm activity through both vacuolar alkalinization and defects in endocytosis [85]. Acridine derivatives like imidazoacridinones (C-1330, C-1415 and C-1558), opens a path as antifungal treatment by its usage in photoantifungal chemotherapy [81,86,87,88,89]. Those compounds were able to enter fungal cells in contrast to C-1311. Imidazoacridinone C-1311, an anti-cancer compound, intercalates into DNA and inhibits human topo II was unable to show antifungal activity against *C. albicans* [70,87,90]. A study conducted by Rzad et al., 2021 showed that conjugation of an imidazoacridinone derivative with octaarginine, bio-active peptide used as nanocarriers to smuggle antimicrobial compounds, emphasizes the penetration of the conjugate into the fungal cells. Authors were able to demonstrate differences in accumulation of both the parent compound and the said conjugate into fungal cells. It resulted in strong antifungal activity, which was correlated with inhibitory effect on yeast topoisomerase II relaxation activity. The effect of conjugated compound was also related with changes in the permeability of the fungal cell membrane. It also showed moderate selectivity for fungal cells over human ones; thus making the conjugate better for anti-fungal rather than anti-cancer therapy [69]. Thus, acridine-peptide conjugates seemed to become a new source of antifungal compounds by exhibiting dual mechanism of action, i.e., killing the microbial cells by membrane disruption on one side and targeting DNA on the other side [69,91,92].

Acridine and its derivatives are considered to have antitumor, antibacterial and antifungal activity due to their broad spectrum of biological activities and mode of action [69,79,80,81,82,83,93,94], including topoisomerase II targeting. Acridine derivative M14 inhibited the fungal hyphae, thus changing the morphology and also showed reduced biofilm formation in *Trichophyton rubrum* and *C. albicans* [95]. Acridine derivatives such as 1-nitro-9-aminoacridine showed antifungal activity against *C. albicans* that are resistant to fluconazole [93]. Acridine conjugates with branched lysine peptides also showed antifungals activity against *C. albicans* [91]. Antifungal activity correlated with inhibitory effect on ScTopo II was also demonstrated for acridine IE6 as well as bis-acridine IE10 derivative. Compound IE6 showed strong antifungal activity even against fluconazole-resistant strains [93]. Moreover, recent studies indicate that acridine derivative Capridine β (C-1748) [86,93,96,97,98] inhibited ScTopo II activity effectively by biotransformation into two metabolites [86]. The reduced form of that compound, named IE1, was responsible for this activity. ScTopo II relaxation activity was inhibited by IE1 at much lower concentrations of 14.1 ± 1.2 µM than by m-AMSA (>200 µM). In contrast, the synthesized form of the reduced metabolite (IE1) did not show antifungal properties in vitro probably due to its inability to reach its molecular target, and to inhibit ScTopo II into fungal cells [86,93].

The antifungal effect of other than acridines, known human topoisomerase II inhibitors was also reported. Studies indicated that aclarubicin showed fungistatic effect for *C. galbrata*, *C. neoformis* and several clinical strains of *C. albicans* [99]. Idarubicin exhibited promising antifungal activity with *A. niger*, *C. glabrata*, *C. neoformis* with MIC in the range of 1.8 and 7.1 µg/mL. Mid-range of antifungal properties along with the morphological changes like predominant growth in yeast form was observed when treated with compounds like daunorubicin, doxorubicin and idarubicin [99], whereas hyphal growth was seen with β-lapachone [100]. These differences in the anthracyclines and β-lapachone are explained by the fact that they act on two different targets. The first set is able to inhibit fungal topoisomerase II and the latter targets on topoisomerase I [70]. Etoposide was able to reduce growth and viability in *C. glabrata* (MIC 5 μg/mL) [101] and *C. dubliniensis* strains (MIC 0.156–0.312 μg/mL) [102]. Moreover, synergistic activity between the topoisomerase II inhibitor and fluconazole for the latter was also reported [102]. Antifungal effect was also described for moxifloxacin—the antibacterial agent belonging to fluoroquinolines and bacterial gyrase and topoisomerase IV-targeting inhibitor [103]. Moxifloxacin was found to be candidacidal in nature and able to affect the yeast to hyphal morphogenesis by disturbing signaling pathways. The compound was also able to arrest the cell cycle of *C. albicans* at S phase. Docking of moxifloxacin with predicted structure of *C. albicans* topo II suggested that moxifloxacin may bind and inhibit the activity of that enzyme in fungal cells [103]. Interestingly, no antifungal activity was observed for other well-known fluoroquinolone antibacterial agents, ciprofloxacin or levofloxacin [104]. Antifungal activity was also reported for eupolauridine, an azafluoranthene alkaloid that targets *S. cerevisiae* by targeting topo II [71].

## 3. Conclusion and Future Perspective

Topoisomerase II activity is essential for fungal cells and can be considered as a promising molecular target for antifungal chemotherapy. The functional significance of differences in fungal and human counterparts with respect of enzyme mode of action or sensitivity to inhibitors is worth decoding. The fact that fungal and mammalian enzymes responded in a different manner to studied compounds suggests that there are sufficient biochemical differences to obtain selectivity for fungi over human cells. To date, mutations were performed on topoisomerase II from *S. cerevisiae* for observing changes in the differential sensitivity towards the various drugs. There is also a need to incorporate mutations at the sites that have major differences in human and fungal enzymes. The effect of mutations can lead to new protein–DNA interactions and thus, new potential fungal topo II-targeting agents could be designed. Drug hypersensitive mutations can also be used for probing drug mechanisms.

Overall, the search for antifungal drug candidates among fungal topoisomerase II inhibitors is undoubtedly worth considering. With better understanding of molecular basis for differences between fungal and human enzyme and increased medicinal chemistry efforts, a new group of antifungals may become a reality in the near future.

## Figures and Tables

**Table 1:** Key amino acid sites and their importance in ScTopo II and human counterparts (HsTopo IIα; HsTopo Iiβ). * Sites of differentiation.

Key Feature	ScTopo II Uniprot P06786	HsTopo Iiα Uniprot P11388	HsTopo Iiβ Uniprot Q02880
ATP Binding site	N70, N99 [34]	N91, N120 [59,60]	N112, N141
Magnesium 1 binding, catalytic	E449, D526 [52]	E461, D541 [61]	E482, D562
Magnesium 2 binding	D526, D528 [52]	D541, D543 [61]	D562, D564 [62]
Transition state stabilizer	R781 [53]	R804	R825
Active site	Y782 [53,63]	Y805	Y826 [62]
Intercalates into and bends DNA	I833 [52]	I856	I877
ATP nucleotide binding	SSN 127–129 GRNGYGAK 140–147 GTK 365–367 [34]	SSN 148–150 GRNGYGAK 161–168 GTK 376–378 [59,60]	SSN 169–171 GRNGYGAK 182–189 GTK 397–399
TOPRIM domain *	C----**L** 443–557	C----**E** 455–572	C----**E** 476–593
A part of K-loop *	KKK 333–336 [51]	KKK 342–344 [51]	KKK 363–365
Interaction with DNA *	K----**N** 965–974 [53]	K----**S** 990–999 [61]	K----**S** 1011–1020

## Data Availability

Not applicable.

## Supplementary Materials

The following supporting information can be downloaded at: https://www.mdpi.com/article/10.3390/molecules27227768/s1. Figure S1. ScTopo II catalytic cycle (1) Enzyme and duplex DNA in relaxed state with n+1 linking number. Dimerization of subunits. (2) Binding and bending of G-segment. (3) Two molecules of ATP binding at closed N-gate. (4) ATP hydrolysis and capture of T-segment. (5) Transient DSB formation in G-segment of DNA-gate and passage of T-strand through it. (6) Re-ligation of G-segment. (7) Opening of C-gate and passage of T-segment and release of 2ADP. (8) Enzyme resetting and representation of duplex DNA with n linking number. Figure S2. Three sequence alignment presented in yeast (ScTopo II) and human topo II isoforms A: Differences in TOPRIM domain (Cys443–Leu557 for ScTopo II; Cys455–Glu572 for HsTopo IIα and Cys476–Glu593 for HsTopo IIβ); B: Region responsible for interaction with DNA (Lys965–Asn974 for ScTopo II; Lys990–Ser999 for HsTopo IIα and Lys1011–Ser1020 for HsTopo IIβ); C: Conserved sequences represented in K-loop region of both yeast and human isoforms. Figure S3: Multiple sequence alignment of three different fungal species *Saccharomyces cerevisiae*, *Candida albicans* and *Candida glabrata* with human topo II isoforms is depicted. Uniprot ID mentioned in the left. The red rectangular bars indicate important consensus sequences like K-loop (333–338 residues), metal bound region (D from 526–528 residues), intercalator region (I at residue 833) and catalytic tyrosine (Y at 782 residue) with respect to *S. cerevisiae*. The differences are marked in blue rectangular bars. Animated graphical representation of yeast topoisomerase II presenting various sites of this enzyme is also provided. The movie was prepared by Molywood [105].
