# Supplementary material for: Targeting DNA Topoisomerase II in Antifungal Chemotherapy
Source: Molecules. 2022 Nov 11;27(22):7768. doi: 10.3390/molecules27227768 (PMC9698242; doi:10.3390/molecules27227768)
Supplement: Supplementary file 1 [file molecules-27-07768-s001.zip › molecules-2005316-supplementary - final .pdf]

molecules

Supplementary Materials

Targeting DNA Topoisomerase II in Antifungal Chemotherapy

Kavya Kondaka and Iwona Gabriel *

Department of Pharmaceutical Technology and Biochemistry, Gdansk University of Technology, 80-233 Gdansk, Poland
* Correspondence: iwogabri@pg.edu.pl; Tel.: +48-58-348-6078; Fax: +48-58-347-1144

Table of contents:

1. **ScTopo II catalytic cycle 1**
2. **Three sequence alignment presented in yeast (ScTopo II) and human topo II isoforms 2**
3. **Multiple sequence alignment of three different fungal species *Saccharomyces cerevisiae*, *Candida albicans* and *Candida glabrata* with human topo II isoforms 3**

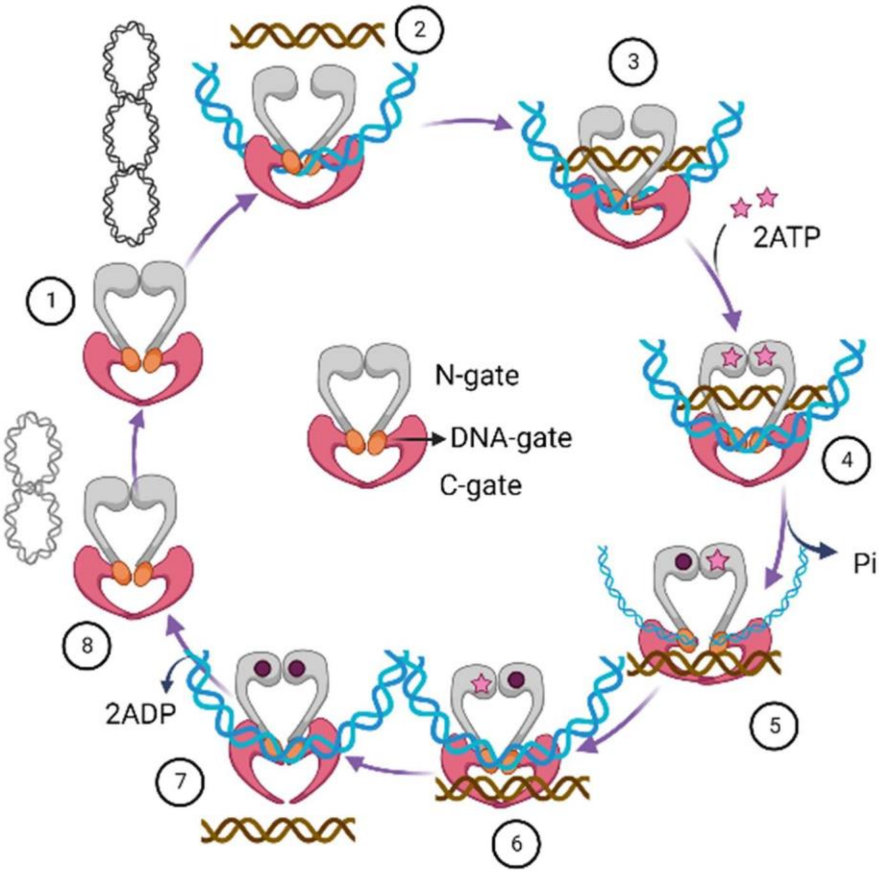

Figure S1. ScTopo II catalytic cycle (1) Enzyme and duplex DNA in relaxed state with n+1 linking number. Dimerization of subunits. (2) Binding and bending of G-segment. (3) Two molecules of ATP binding at closed N-gate. (4) ATP hydrolysis and capture of T-segment. (5) Transient DSB formation in G-segment of DNA-gate and passage of T-strand through it. (6) Re-ligation of G-segment. (7) Opening of C-gate and passage of T-segment and release of 2ADP. (8) Enzyme resetting and representation of duplex DNA with n linking number. Created with BioRender.com

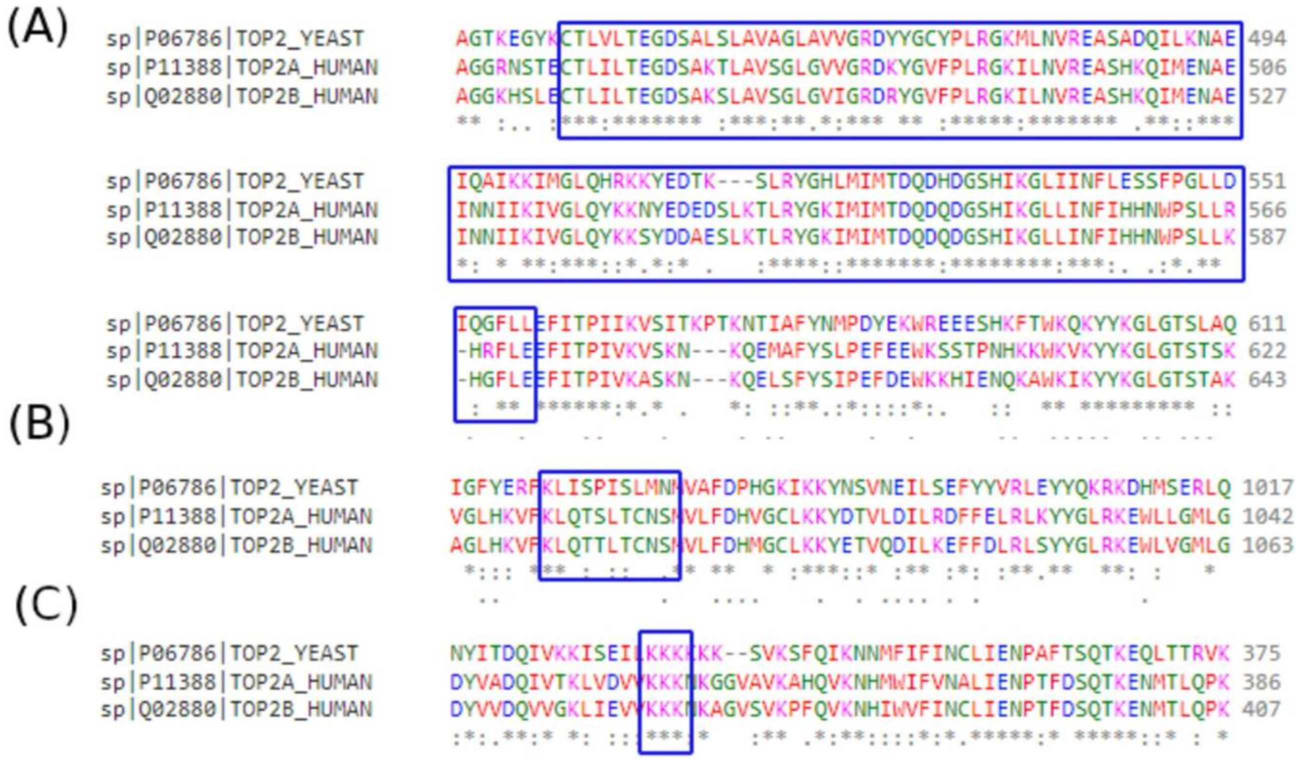

Figure S2. Three sequence alignment presented in yeast (ScTopo II) and human topo II isoforms A: Differences in TOPRIM domain (Cys443 – Leu557 for ScTopo II; Cys455 – Glu572 for HsTopo II*α* and Cys476 – Glu593 for HsTopo IIβ) ; B: Region responsible for interaction with DNA (Lys965 – Asn974 for ScTopo II; Lys990 – Ser999 for HsTopo II*α* and Lys1011 – Ser1020 for HsTopo IIβ); C: Conserved sequences represented in K-loop region of both yeast and human isoforms.

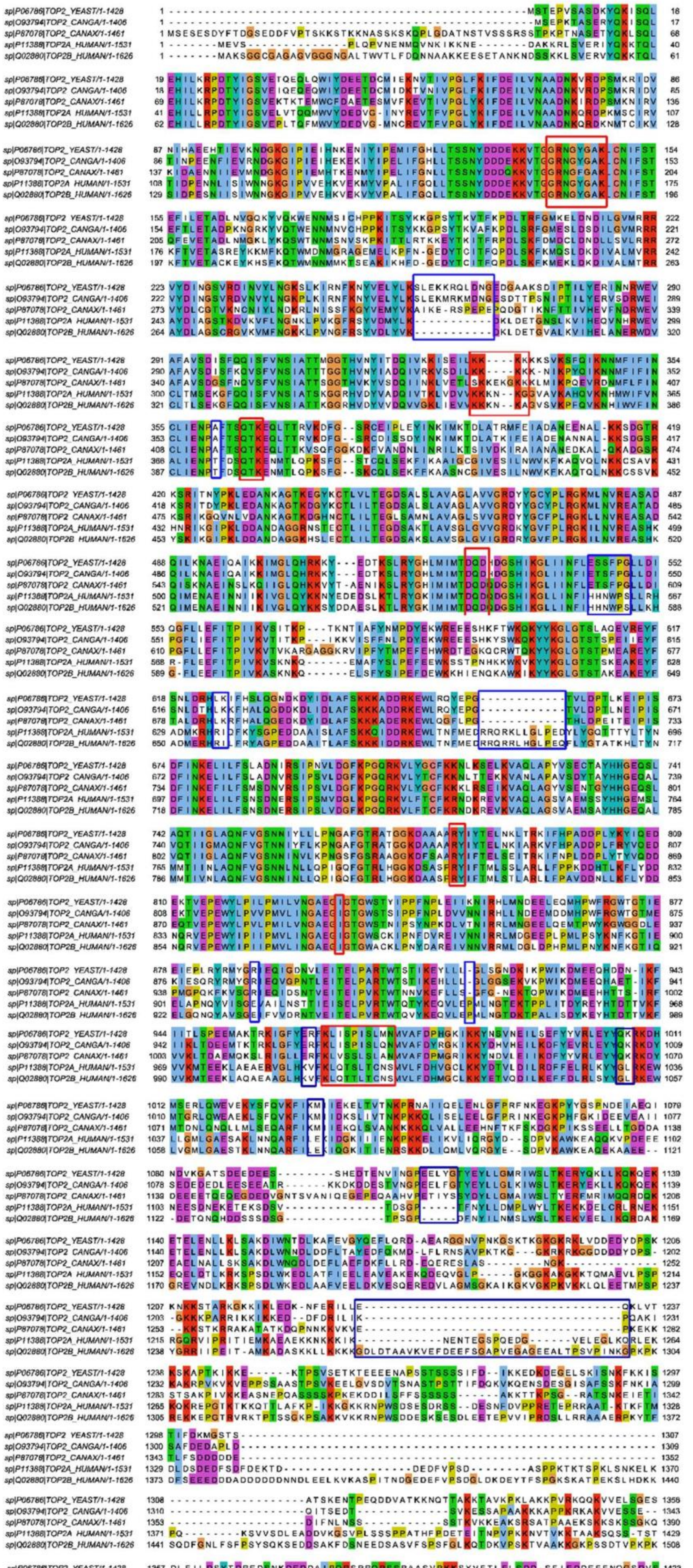
sp|P06786|TOP2_YEAST/1-1428
sp|Q93794|TOP2_CANGA/1-1406
sp|P87078|TOP2_CANAX/1-1461
sp|P11388|TOP2A_HUMAN/1-1531
sp|Q02880|TOP2B_HUMAN/1-1626

Figure S3: Multiple sequence alignment of three different fungal species *Saccharomyces cerevisiae*, *Candida albicans* and *Candida glabrata* with human topo II isoforms is depicted. Uniprot ID mentioned in the left. The red rectangular bars indicate important consensus sequences like K-loop (333–338 residues), metal bound region (D from 526–528 residues), intercalator region (I at residue 833) and catalytic tyrosine (Y at 782 residue) with respect to S. cerevisiae. The differences are marked in blue rectangular bars.
